# Albumin-mediated extracellular zinc speciation drives cellular zinc uptake†

**DOI:** 10.1039/d2cc02278h

**Published:** 2022-06-02

**Authors:** James P. C. Coverdale, Hugo A. van den Berg, Siavash Khazaipoul, Hannah E. Bridgewater, Alan J. Stewart, Claudia A. Blindauer

**Affiliations:** Department of Chemistry, University of Warwick Coventry CV4 7AL UK c.blindauer@warwick.ac.uk; School of Pharmacy, Institute of Clinical Sciences, University of Birmingham Edgbaston B15 2TT UK; Warwick Mathematics Institute, University of Warwick Coventry CV4 7AL UK; School of Medicine, University of St. Andrews St. Andrews KY16 9TF UK

## Abstract

The role of the extracellular medium in influencing metal uptake into cells has not been described quantitatively. In a chemically-defined model system containing albumin, zinc influx into endothelial cells correlates with the extracellular free zinc concentration. Allosteric inhibition of zinc-binding to albumin by free fatty acids increased zinc flux.

## Abstract 2

Fatty acids alter zinc speciation in plasma, increasing zinc influx into endothelial cells.

## Graphical abstract

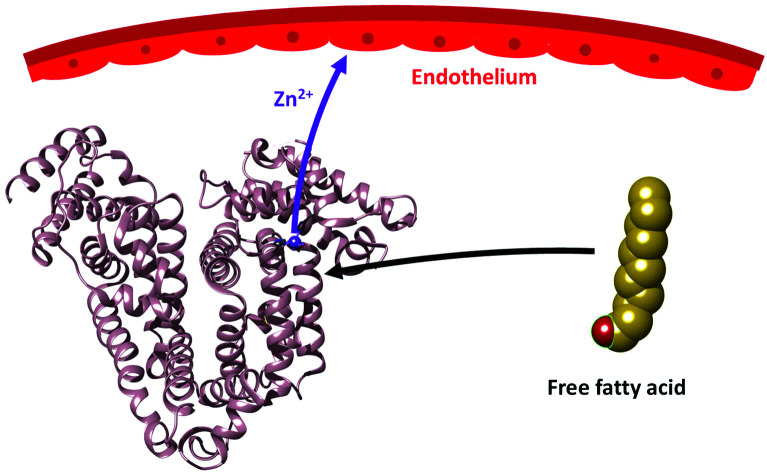

Zinc is, after iron, the second-most abundant essential d-block metal in the body. Zn^2+^ is found in almost all cells, with total cytosolic concentrations in the hundreds of micromolar.^1^ In mammals, Zn^2+^ is distributed throughout the body by blood plasma,^2^ where total concentrations are around 10–23 μM under normal physiological conditions.^3^ Transport from plasma into tissues requires Zn^2+^ uptake into cells, and although these total concentrations may suggest that zinc import occurs against a concentration gradient, this view is inappropriate, as it is the concentration of the “active” species – presumably the free Zn^2+^ ion or its simple small-molecule complexes – that needs to be considered.^4^ Free metal concentrations are a consequence of binding affinities and concentrations of ligands in the respective solution, and here, the tables are turned, with non-protein bound Zn^2+^ estimated to lie in the (high) picomolar range in the cytosol,^1^ and at least one order of magnitude higher in blood plasma.^5–7^ Free Zn^2+^ above low nanomolar concentration is in fact remarkably toxic towards cells of all species including mammalian,^8^ plant,^9^ fungal and certain bacterial cells,^10^ and so careful control of zinc homeostasis – also in the extracellular environment – is paramount to survival and health.

In mammals, serum albumin is the major carrier for Zn^2+^ in plasma.^11^ Albumins from several species bind Zn^2+^ at two principal binding sites: site A and site B.^12–16^ Site A, also called the ‘multi-metal binding site’,^13^ is an interdomain site between domains I and II (Fig. 1, left).^15,16^ X-Ray crystallography of human (HSA) and equine serum albumins has shown tetrahedral coordination of Zn^2+^ by three amino acid residues (His67, His247 and Asp249) and a fourth solvent site.^15^

**Fig. 1:**
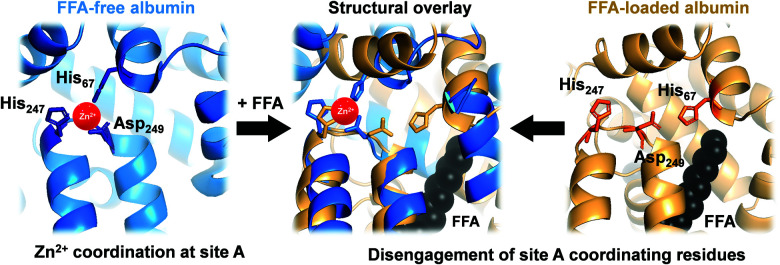
Serum albumin is the principal Zn^2+^ transporter in the extracellular space. Amino acid residues His67, His247 and Asp249 form site A (blue; PDB 5IJF; HSA), the principlal interdomain binding site for Zn^2+^ (red sphere). In the presence of FFAs of sufficient chain length bound at the nearby FFA binding site FA2 (myristate C14:0 shown in black), site A residues disengage, as depicted in the structural overlay (centre), and Zn^2+^ affinity drops dramatically,^17,18^ leading to release of Zn^2+^ from FFA-loaded albumin (tan; PDB 1BJ5; HSA^30^) under physiological conditions.^32^

Owing to albumin's high concentration (>600 μM in human plasma), moderate affinity for Zn^2+^ (site A displays a low micromolar dissociation constant under physiological conditions),^17–20^ and the labile fashion in which Zn^2+^ is bound, this site is responsible for the largest portion of the exchangeable plasma zinc pool. Thus, albumin acts as the major determinant of zinc speciation in plasma.^8,21^ Plasma zinc exchanges about 150 times per day.^2^ This illustrates the highly dynamic character of zinc speciation in plasma – not least because several processes occurring within plasma are zinc-mediated, including blood clotting, insulin activation, and immune function.^22–24^ One particular group of metabolites has a major impact on albumin-mediated zinc speciation: free (*i.e.*, non-esterified) fatty acids (FFA). FFA levels in plasma are also highly dynamic, and are significantly elevated under certain physiological (*e.g.* fasting) and pathological (diabetes, obesity) conditions.^25,26^ Several fatty acid binding sites have been identified in crystallographic and NMR spectroscopic studies of albumin.^27,28^ FFA binding to the high-affinity FA2 site (Fig. 1, right) is thought to be a key factor in eliciting a structural change to the domain I/II interface, and hence in displacing His67 (domain I) from the vicinity of His247 and Asp249 (domain II) (Fig. 1, centre). This interaction depends on the nature of the FFA, including its length, whereby only medium-to-long chain fatty acids (>C12) cause the allosteric switch.^29,30^ Isothermal titration calorimetry of bovine serum albumin (BSA) and HSA has demonstrated that this dramatically decreases their Zn^2+^-binding capacity.^14,17,25,29,31^ We recently quantified changes in Zn^2+^ speciation in bovine serum and human plasma resulting from elevated free fatty acid (FFA) concentrations, and found that a re-distribution of Zn^2+^ from albumin (BSA or HSA) to other proteins occurs.^32^ More recently, we have shown that FFAs affect zinc-mediated processes such as platelet aggregation and fibrin clotting *via* albumin.^29^ Both processes depend on the concentration of Zn^2+^ that is available to bind to the proteins involved.

The present study is based on the hypothesis that previously observed effects of albumin-mediated zinc speciation on cells^8^ are due to more “free” Zn^2+^ becoming available to membrane-bound transporters such as ZIPs,^33^ which would result in increased zinc uptake. We test this hypothesis by quantitatively assessing Zn^2+^ influx (*φ*_in_) and efflux (*φ*_out_) rates in model systems with well-defined concentrations of BSA and Zn^2+^. Moreover, we assess the effect of FFAs (octanoate, myristate and palmitate) on flux rates, and whether this affects zinc toxicity.

Zinc binding in both BSA and HSA is affected similarly by FFA binding,^31^ so BSA may serve as an appropriate model for mammalian albumins. Furthermore, the widespread use of foetal bovine serum in media for culturing human cell lines highlights the relevance of BSA-based model systems. Indeed, the importance of zinc buffering and speciation in cell culture media in general,^34^ and by albumin in particular^8,35^ has been demonstrated in recent years.

We selected a human umbilical vascular endothelial cell (HUVEC) model, as *in vivo*, vascular endothelial cells are in direct contact with blood plasma and form a barrier through which all nutrients, including Zn^2+^, must pass prior to entry into peripheral tissues. Endothelial cell models thus provide a well-defined system that is pertinent for assessing the role(s) that albumin plays in cellular zinc uptake. Primary HUVECs were immortalised using human telomerase reverse transcriptase (hTERT) to provide a homologous cell line for further study (ESI,† Materials and methods). The resultant cells exhibited a typical endothelial cell morphology in culture and retained expression of HUVEC markers including CD31, von Willebrand factor, and VE-cadherin (ESI,† Fig. S1).

For the determination of zinc flux rates, we developed a stable isotope approach. Traditionally, zinc dynamics have often been assessed using radioactive ^65^Zn. This comes with several drawbacks, including the fact that ^65^Zn decays to ^65^Cu, which introduces another chemical element that may affect cells. A recent study highlighted the merits of working with stable isotopes.^36^

Before commencement of the isotope assay, immortalised HUVECs were first equilibrated to physiologically relevant conditions (600 μM BSA and 20 μM natural-abundance Zn^2+^ (designated as “pre-conditioning medium”, Fig. 2) for 24 h. After this time, cells were washed with phosphate-buffered saline and incubated in fresh medium containing 20 μM ^68^Zn^2+^ (>99% ^68^Zn; ESI,† Table S1) with different extracellular BSA concentrations (0–600 μM). Cell pellets were collected at defined time intervals, digested with ultrapure concentrated nitric acid, and the intracellular isotopic ratio ^66^Zn/^68^Zn was measured by standard inductively-coupled plasma-mass spectrometry (ICP-MS) (Fig. 2(a)). Additionally, the total amount of intracellular Zn^2+^ (*Q*_0_ and *Q*(*t*)) was determined from the sum of all zinc isotopes (Fig. 2(b)).

**Fig. 2:**
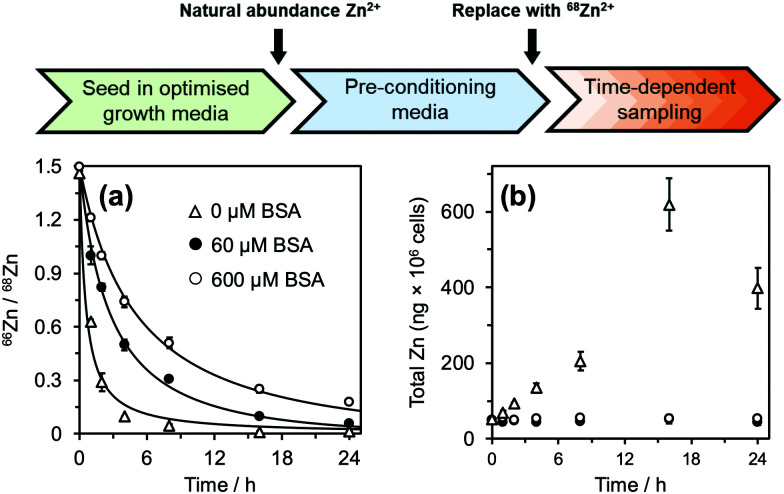
^66^Zn/^68^Zn ratios determined at different extracellular albumin concentrations (0–600 μM; see ESI† for full data). Phase I: HUVEC growth medium. Phase II: Physiologically-relevant media (600 μM BSA + 20 μM natural abundance Zn^2+^, no FFA). Phase III: Media containing variable concentration of BSA (0-600 μM) + 20 μM isotopically-enriched ^68^Zn^2+^, with/without FFA supplementation. Cell pellets were collected in a time-dependent manner and analysed by ICP-MS. (a) ^66^Zn/^68^Zn ratios were calculated, plotted and fitted to a mathematical model to derive zinc flux rates. Zinc flux increases with decreasing BSA concentration. (b) Zinc isotopes (^64^Zn, ^66^Zn, ^67^Zn, ^68^Zn, ^70^Zn) were summed up to determine total intracellular zinc (ng × 10^6^ cells). For extracellular [BSA] > 60 μM, intracellular [Zn] remained constant at *ca.* 50 ng × 10^6^ cells but increased for extracellular [BSA] < 40 μM. See ESI,† Tables S2–S5 and S9–S13 for full numerical data and mathematical models.

For cells treated with 60–600 μM BSA, the total intracellular [Zn] remained constant (∼50 ng per million cells: Fig. 2(b) and ESI,† Tables S2 and S3). This indicates that the cells are able to control their total contents, and that rates of influx (*φ*_in_) and efflux (*φ*_out_) are equal, *φ*_in_ = *φ*_out_. In contrast, cells in the presence of lower concentrations of BSA (0 and 40 μM) accumulated zinc over time (Fig. 2(b) and ESI,† Tables S2 and S3), *i.e.*, influx rates are larger than efflux rates; *φ*_in_ > *φ*_out_.

We sought to derive quantitative information on zinc fluxes from these data, and developed a mathematical model to describe the ^66^Zn/^68^Zn ratio as a function of time:1
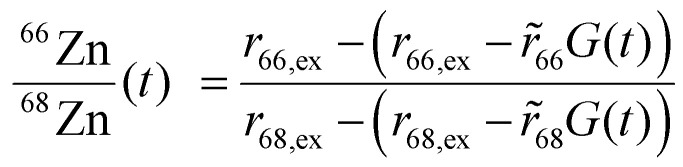
where *r*_66,ex_ (or *r*_68,ex_) is the relative abundance of ^66^Zn (or ^68^Zn) in the incubation medium, *r̃*_66_ or *r̃*_68_ is the natural relative abundance of ^66^Zn or ^68^Zn, and *G*(*t*) is a function given by2
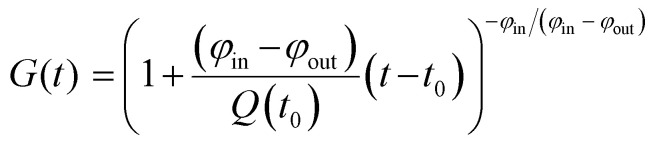
where *t*_0_ denotes the point in time at which the experiment is started, *Q*(*t*_0_) the total cellular zinc content at time *t* = *t*_0_, *φ*_in_ the influx, and *φ*_out_ the efflux of zinc. This relationship is based on the assumption that both fluxes are constant for the duration of the experiment (see ESI,† for validation). In cases where cells do not accumulate zinc over time *Q*(*t*) ≡ *Q*_0_, and hence *φ*_in_ = *φ*_out_ ≡ *φ*), *G*(*t*) reduces to

3 *G*(*t*) = exp{(−*φ*/*Q*_0_)(*t* − *t*_0_)}.

The datasets for 60, 160 and 600 μM BSA were treated using the latter relationship; those at 0 and 40 μM BSA used eqn (2). Representative fits are shown in Fig. 2, all fitting results are collected in ESI,† Fig. S2 and Tables S4, S5 and S9–S13. The zinc influx rates obtained (2.51–22.82 fg h^−1^ cell^−1^) are comparable to those found in previous studies. An early radio-chemical study determined ^65^Zn^2+^ influx rates into human and rat erythrocytes of 0.22 fg h^−1^ cell^−1^,^37^ while an ICP-MS study of ^70^Zn^2+^ influx into human HEK293T cells in the presence of Zn-depleted media determined rates of 23.5 fg h^−1^ cell^−1^.^36^

Plotting the influx rates *vs.* total [BSA] clearly shows that cellular zinc uptake rates are dependent on albumin concentration (Fig. 3(a)). This can be rationalised on the basis that BSA controls zinc speciation, and that free [Zn^2+^] governs interaction with membrane-bound zinc transporters – and hence transport. This is clearly seen in Fig. 3(b), where we have estimated free [Zn^2+^], based on published stability constants for site A,^19,31^ and replotted the observed influx rates *vs.* these. This plot suggests that in this low concentration range, transport kinetics are 1^st^ order in free [Zn^2+^], in agreement with expectations for transport of single species across membranes^38^ and previous findings in hepatocytes,^39^ as well as with kinetic data for a bacterial ZIP protein.^40^

**Fig. 3:**
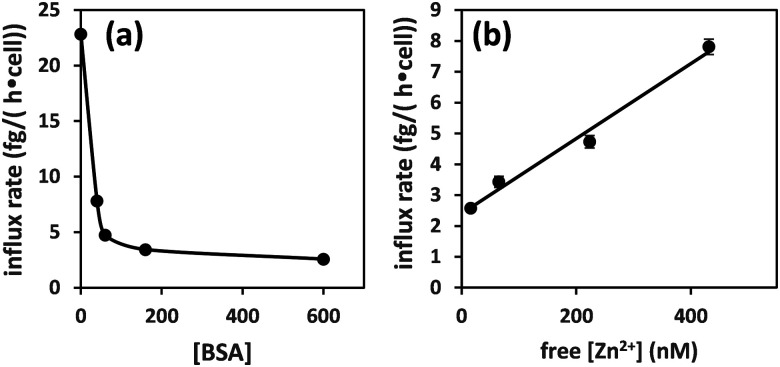
Zinc influx depends on the concentration of albumin (a). This can be correlated to free [Zn^2+^] for [BSA] = 40–600 μM (estimated using published stability constants for site A only; see ESI†) (b). The influx rate shows a linear relationship with free [Zn^2+^].

Having thus found that, in systems where BSA is the only significant zinc buffer, this protein governs zinc uptake rates *via* regulating free [Zn^2+^], we explored next the impact of FFAs on zinc uptake. Previous work has shown that 5 molar equivalents of long-chain FFAs (levels that can be encountered in certain pathophysiological conditions)^28^ had a pronounced effect on site A availability.^17,29,31^ For these experiments, we chose a BSA concentration of 60 μM, for several reasons: (i) at 60 μM fatty-acid free BSA, cellular zinc contents remained stable, (ii) a solution that contains 10% of physiological levels is reminiscent of cell culture media, and (iii) long-chain FFAs are sparingly soluble in neutral aqueous solutions, so achieving concentrations that would be high enough to match those required for 600 μM BSA is experimentally challenging. Thus, the medium for time-dependent sampling contained 60 μM BSA, 20 μM Zn^2+^, and was supplemented with 300 μM octanoate (C8:0), myristate (C14:0), or palmitate (C16:0).

Addition of myristate or palmitate significantly increased the rate of intracellular ^68^Zn isotope enrichment, whilst octanoate had no discernible effect (Fig. 4 and ESI,† Tables S5, S7). Furthermore, the increased change in the ^66^Zn/^68^Zn ratio observed in the presence of myristate or palmitate was accompanied by a gradual increase in total intracellular zinc over time, indicating that cells were no longer able to balance influx and efflux (ESI,† Tables S3 and S6). Accordingly, fitting the data to our model (eqn (1) and (2)) gave *φ*_in_ = 7.45 ± 0.34 in the presence of myristate or 7.99 ± 0.24 fg h^−1^ cell^−1^ in the presence of palmitate, compared to 4.73 ± 0.20 fg h^−1^ cell^−1^ in the absence of FFA. In contrast, the rate for BSA treated with octanoate (5.03 ± 0.31 fg h^−1^ cell^−1^) remained statistically unchanged relative to the FFA-free experiment (Fig. 4 and ESI,† Table S5). These observations agree with earlier chromatographic, NMR and ITC studies, which suggest that octanoate is too short to activate the allosteric switch.^17,29,31,32^

**Fig. 4:**
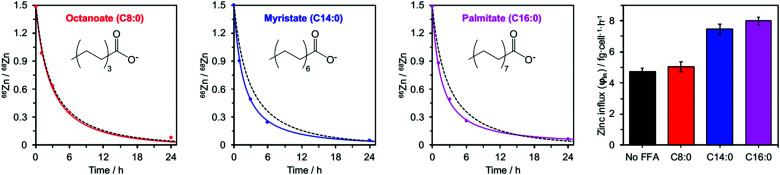
Isotopic ratios (^66^Zn/^68^Zn) over time for HUVEC cells cultured in presence of 60 μM BSA and either C8:0 (octanoate), C14:0 (myristate) or C16:0 (palmitate) FFAs (300 μM, 5 mol. equiv.). Experimental data (•) are shown with corresponding fitting model for that experimental condition (solid coloured line) alongside the fitting model for 60 μM BSA in the absence of FFAs (dashed black line). The right-hand panel compares the influx rates *φ*_in_ for these four conditions. Full numerical data can be found in ESI,† Tables S6, S7 and S14–S16.

Finally, to test whether zinc speciation and the resulting increased influx affects zinc toxicity, we determined IC_50_ values in cells grown at different [BSA] (ESI,† Table S8 and Fig. S3). At 600 μM BSA, an IC_50_ value of 1025 ± 12 μM Zn^2+^ was found, contrasting with 11.80 ± 0.05 μM in the absence of BSA. The increased influx observed in presence of long-chain FFAs was also accompanied by increased toxicity, with IC_50_ dropping from 111 ± 2 μM to 66.7 ± 0.9 μM for experiments at 60 μM BSA in the absence and presence of myristate, respectively. These results indicate that increased influx is accompanied by measurable effects on cells.

In summary, we have developed an integrated quantitative approach that permits the determination of zinc flux rates in (endothelial-like) cells. Supplying a single stable isotope in the extracellular medium, monitoring isotopic ratios over time, and mathematical modelling allows assessing zinc uptake under essentially physiological conditions. Crucially, our approach does neither require the cells to accumulate more zinc than their physiological quota, nor the administration of any additional reagents.

Our findings provide the first directly quantifiable evidence that (i) extracellular zinc speciation governs zinc uptake rates, and (ii) FFAs may affect cellular zinc uptake, *via* the allosteric switch on albumin. This may imply that *in vivo* acute and chronic elevations of plasma FFAs drive increased export of Zn^2+^ from plasma. We note however that at physiological albumin concentrations (*ca.* 600 μM), total cellular zinc contents would not be expected to increase like for [BSA] = 60 μM. Future efforts should evaluate zinc flux rates in more complex, multi-protein systems, and may involve refinements of isotope measurements and mathematical models.

This work was financially supported by the Leverhulme Trust (RPG-2017-214) and BBSRC (BB/J006467/1 and BB/V014684/1). We thank Prof. Andrew Riches (University of St. Andrews) for provision of materials, and Dr Elizabeth Bolitho (University of Warwick) for assistance with cell culture experiments.

## Conflicts of interest

There are no conflicts to declare.

## Supplementary Material

CC-058-D2CC02278H-s001
