# Supplementary material for: Albumin-mediated extracellular zinc speciation drives cellular zinc uptake
Source: Chem Commun (Camb). 2022 Jun 2;58(53):7384–7. doi: 10.1039/d2cc02278h (PMC9244874; doi:10.1039/d2cc02278h)
Supplement: CC-058-D2CC02278H-s001 [file CC-058-D2CC02278H-s001.pdf]

Electronic Supplementary Material (ESI) for ChemComm.
This journal is © The Royal Society of Chemistry 2022

Fatty acids modulate zinc uptake into cells via albumin binding

James P. C. Coverdale, Hugo A. van den Berg, Siavash Khazaipoul, Hannah E. Bridgewater, Alan J. Stewart, and Claudia. A. Blindauer

Supplementary Information

Supplemental Tables S1-S16

Supplemental Figures S1-S3

Materials and Methods

Derivation of the Mathematical Model

Table S1. Isotopic purity of ^{68}Zn stock solutions (500 µM in 1× HEPES-buffered EBSS), determined by ICP-MS in He-gas mode. These isotopic abundances were considered in mathematical modelling of experimental data.

Analyte	Abundance (%)
^{64}Zn	0.08
^{66}Zn	0.05
^{67}Zn	0.33
^{68}Zn	99.33
^{70}Zn	0.21

Table S2. Total intracellular Zn (fg·cell^{-1}) determined for the intracellular Zn concentration in immortalised HUVEC cells treated with isotopically enriched $^{68}Zn^{2+}$ (20 µM solution in EBSS, pH 7.4) for defined time period (*t*) with defined supplementation using bovine serum albumin (BSA; 0-0.6 mM), after 24 h pre-incubation under physiological conditions (20 µM natural abundance Zn^{2+}, 0.6 mM BSA solution in EBSS, pH 7.4).

Total intracellular Zn (fg·cell^{-1})					
**Time / h**	**0 µM BSA**	**40 µM BSA**	**60 µM BSA**	**160 µM BSA**	**600 µM BSA**
0	52 ± 4	52 ± 2	50 ± 1	52 ± 2	50 ± 2
0.08 (5 min)	54 ± 4	-	-	-	-
0.17 (10 min)	56 ± 2	-	-	-	-
0.33 (20 min)	55.9 ± 0.5	-	-	-	-
0.5 (30 min)	60 ± 9	-	-	-	-
0.67 (40 min)	60± 4	-	-	-	-
0.83 (50 min)	61 ± 5	-	-	-	-
1	68 ± 7	64 ± 3	45 ± 3	55 ± 1	56 ± 3
2	93 ± 2	-	48 ± 1	-	50 ± 4
3	-	70 ± 4	-	50 ± 6	-
4	135 ± 11	-	44 ± 5	-	54 ± 3
6	-	81 ± 5	-	48 ± 2	-
8	205 ± 25	-	46 ± 3	-	56 ± 5
16	619 ± 69	-	52 ± 12	-	54 ± 6
24	398 ± 54	110 ± 12	45 ± 8	53 ± 5	53 ± 2

Table S3. P-values testing whether immortalised HUVEC cells significantly accumulate zinc, using linear regression alongside calculated slopes (fg·cell^{-1}·h^{-1}) and calculated standard error. Probabilities shown relate to experimental data reported in either **Table S2** (variable bovine serum albumin concentration 0-600 µM, no FFA) or **Table S5** (FFAs supplemented at 300 µM, 5 mol. equiv., with BSA concentration fixed at 60 µM).

BSA / µM	FFA	Slope / fg·cell^{-1}·h^{-1} (standard error)	P-value
0	-	20.4266 (3.27769)	0.000065
40	-	2.14023 (0.351819)	0.0089
60	-	0.00854449 (0.14761)	0.96
60	C8:0 (5 mol. equiv.)	0.0117707 (0.0312627)	0.73
60	C14:0 (5 mol. equiv.)	1.31218 (0.226061)	0.010
60	C16:0 (5 mol. equiv.)	1.45548 (0.262727)	0.012
160	-	0.0271238 (0.157038)	0.87
600	-	0.0521509 (0.122045)	0.69

Table S4. Isotopic ratios ($^{66}Zn/^{68}Zn$) determined for the intracellular Zn concentration in immortalised HUVEC cells treated with isotopically enriched $^{68}Zn^{2+}$ (20 µM solution in EBSS, pH 7.4) for defined time period (*t*) with defined supplementation using BSA (0-0.6 mM), after 24 h pre-incubation under physiological conditions (20 µM natural abundance Zn^{2+}, 0.6 mM BSA solution in EBSS, pH 7.4).

Intracellular Zn isotopic ratio ($^{66}Zn/^{68}Zn$)					
**Time / h**	**0 µM BSA**	**40 µM BSA**	**60 µM BSA**	**160 µM BSA**	**600 µM BSA**
0	1.462 ± 0.009	1.514 ± 0.003	1.501 ± 0.005	1.514 ± 0.003	1.495 ± 0.006
0.08 (5 min)	1.39 ± 0.02	-	-	-	-
0.17 (10 min)	1.29 ± 0.03	-	-	-	-
0.33 (20 min)	1.20 ± 0.02	-	-	-	-
0.5 (30 min)	1.05 ± 0.02	-	-	-	-
0.67 (40 min)	1.0 ± 0.2	-	-	-	-
0.83 (50 min)	0.771 ± 0.008	-	-	-	-
1	0.63 ± 0.01	0.89 ± 0.04	1.00 ± 0.05	1.1 ± 0.2	1.21 ± 0.02
2	0.29 ± 0.05	-	0.82 ± 0.02	-	1.00 ± 0.02
3	-	0.47 ± 0.02	-	0.8 ± 0.1	-
4	0.097 ± 0.003	-	0.50 ± 0.03	-	0.74 ± 0.03
6	-	0.27 ± 0.01	-	0.47 ± 0.06	-
8	0.047 ± 0.006	-	0.308 ± 0.003	-	0.51 ± 0.03
16	0.011 ± 0.002	-	0.099 ± 0.003	-	0.25 ± 0.02
24	0.012 ± 0.002	0.046 ± 0.003	0.059 ± 0.006	0.105 ± 0.002	0.18 ± 0.01

Table S5. Mathematical modelling estimates for zinc flux rates (Φ_{in} and Φ_{out}, fg·cell^{-1}·h^{-1}) as a function of extracellular [BSA] and supplementation of free fatty acids (FFAs). The total intracellular zinc concentration (*Q*(*t*))(**ESI† Tables S2, S3** and **S5**) dictated the modelling approach.[a,b]

			**Zinc flux (fg·cell^{-1}·h^{-1})**	
**[BSA] (μM)**	**FFA**	**Constant *Q*(*t*)?**	φ_{in}	φ_{out}
0	-	No	22.82 ± 2.61	2.39 [c]
40	-	No	7.81 ± 0.25	3.54 ± 0.86
60	-	Yes	4.73 ± 0.20	4.73 ± 0.20
160	-	Yes	3.43 ± 0.18	3.43 ± 0.18
600	-	Yes	2.57 ± 0.09	2.57 ± 0.09
60	C8:0	Yes	5.03 ± 0.31	5.03 ± 0.31
60	C14:0	No	7.45 ± 0.34	4.64 ± 0.78
60	C16:0	No	7.99 ± 0.24	2.51 ± 1.22

[a] Uncertainties in columns 4 and 5 refer to standard errors.

[b] Comparison across all data sets (except [BSA] = 0 μM) showed that the variation of φ_{in} was highly significant ($F\{2,29\} = 4.26$, $P = 1.15 \cdot 10^{-16}$) whereas the variation of φ_{out} was not significant at the 1% level ($F\{2,29\} = 4.26$, $P = 0.024$).

[c] $\varphi_{in} - \varphi_{out}$ was constrained by the slope of accumulation (20.43) (**ESI† Table S3**)

Table S6. Total intracellular Zn (fg·cell^{-1}) determined for the intracellular Zn concentration in immortalised HUVEC cells treated with isotopically enriched $^{68}Zn^{2+}$ (20 µM) for defined time period (*t*) with defined supplementation using BSA (60 µM) + 5 mol. equiv. FFA (octanoate, myristate, or palmitate), after 24 h pre-incubation under physiological conditions (20 µM natural abundance Zn^{2+}, 600 µM BSA solution in EBSS, pH 7.4).

Total intracellular Zn (fg·cell^{-1})			
**Time (t) / h**	**Octanoate (C8:0)**	**Myristate (C14:0)**	**Palmitate (C16:0)**
0	50 ± 6	55 ± 6	52 ± 9
1	50 ± 5	61 ± 4	56 ± 14
3	49 ± 5	61 ± 6	64 ± 8
6	49 ± 3	73 ± 9	72 ± 5
24	50 ± 2	89 ± 7	90 ± 18

Table S7. Isotopic ratios ($^{66}Zn/^{68}Zn$) determined for the intracellular Zn concentration in immortalised HUVEC cells treated with isotopically enriched $^{68}Zn^{2+}$ (20 µM) for defined time period (*t*) with defined supplementation using BSA(60 µM) + 5 mol. equiv. FFA (octanoate, myristate, or palmitate), after 24 h pre-incubation under physiological conditions (20 µM natural abundance Zn^{2+}, 600 µM BSA BSA solution in EBSS, pH 7.4).

Intracellular Zn isotopic ratio ($^{66}Zn/^{68}Zn$)			
**Time (t) / h**	**Octanoate (C8:0)**	**Myristate (C14:0)**	**Palmitate (C16:0)**
0	1.480 ± 0.006	1.503 ± 0.005	1.480 ± 0.006
1	0.98 ± 0.04	0.90 ± 0.02	0.88 ± 0.03
3	0.63 ± 0.04	0.49 ± 0.01	0.49 ± 0.01
6	0.35 ± 0.02	0.2442 ± 0.0002	0.259 ± 0.002
24	0.080 ± 0.002	0.047 ± 0.001	0.064 ± 0.008

Table S8. IC_{50} concentrations for Zn^{2+} (administered as $ZnCl_2$) determined in immortalised HUVEC cells in the presence of BSA (0-600 μM) with defined supplementation with free fatty acids (FFAs): either none, C8:0 (as sodium octanoate), or C14:0 (as sodium myristate). P-values for the latter two conditions refer to the comparison to the fatty acid-free control at 60 μM BSA and were calculated using a two-tailed *t*-test assuming unequal variances (Welch's *t*-test). Data are shown graphically in **Figure S3**.[a)]

BSA / μM	FFA	IC_{50} / μM
0	-	11.80 ± 0.05
6	-	17.4 ± 0.1
30	-	50.4 ± 0.4
60	-	111 ± 2
60	C8:0 (300 μM, 5 mol. equiv.)	108 ± 3 (p=0.2452)
60	C14:0 (300 μM, 5 mol. equiv.)	66.7 ± 0.9 (p=0.0008) ***
600	-	1025 ± 12

[a)] Using the datapoints at 0, 30, 60 and 600 μM BSA, assuming that in all cases, free [Zn^{2+}] = 11.80 μM (corresponding to the IC_{50} for free [Zn^{2+}], and with the approximations that (i) at excess zinc, site A is fully occupied, and (ii) the remainder of binding is due to site B, it is possible to estimate the conditional stability constant for site B as log K' = 5.0±0.4.

Tables S9-S16. Mathematical modelling results (data fits shown in Figure S3). Note that in cases where only the parameter ϕ_{in} is given, ϕ_{out} is equal to ϕ_{in}, as no change in total intracellular Zn (*Q*) occurred.

Table S9: 0 μM BSA*				
	**Estimate**	**Standard Error**	**t-Statistic**	**P-value**
ϕ_{in}	20.4266	3.27769	6.232	0.0000642045
ϕ_{out}	2.39 *	-	-	-

Table S10: 40 μM BSA				
	**Estimate**	**Standard Error**	**t-Statistic**	**P-value**
ϕ_{in}	7.81046	0.245476	31.8176	0.000068
ϕ_{out}	3.53528	0.855783	4.13105	0.026

Table S11: 60 μM BSA				
	**Estimate**	**Standard Error**	**t-Statistic**	**P-value**
ϕ_{in}	4.73129	0.199874	23.6713	3.73×10^{-7}

Table S12: 160 μM BSA				
	**Estimate**	**Standard Error**	**t-Statistic**	**P-value**
ϕ_{in}	3.43565	0.18388	18.6843	0.000048

Table S13: 600 μM BSA				
	**Estimate**	**Standard Error**	**t-Statistic**	**P-value**
ϕ_{in}	2.56974	0.0906118	28.3599	1.27×10^{-7}

Table S14: 60 μM BSA + sodium octanoate (C8:0, 0.3 mM, 5 mol. equiv.)				
**Table S13**	**Estimate**	**Standard Error**	**t-Statistic**	**P-value**
ϕ_{in}	5.03793	0.30771	16.3723	0.000081

Table S15: 60 μM BSA + sodium myristate (C14:0, 0.3 mM, 5 mol. equiv.)				
	**Estimate**	**Standard Error**	**t-Statistic**	**P-value**
ϕ_{in}	7.4492	0.230988	32.2493	0.000066
ϕ_{out}	4.6454	0.775272	5.99196	0.0093

Table S16: 60 μM BSA + sodium palmitate (C16:0, 0.3 mM, 5 mol. equiv.)				
	**Estimate**	**Standard Error**	**t-Statistic**	**P-value**
ϕ_{in}	7.98621	0.336722	23.7175	0.00016
ϕ_{out}	2.50888	1.21877	2.05853	0.13

* ϕ_{out} estimated from difference between slope (Table S3) and ϕ_{in}.

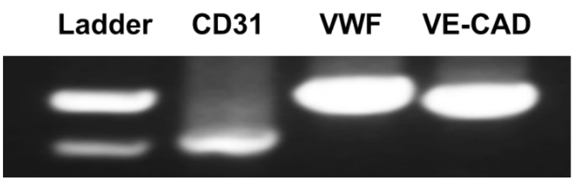

Figure S1. mRNA expression of endothelial marker proteins in immortalised HUVECs; CD31, von Willebrand factor (vWF) and VE-cadherin (VE-CAD).

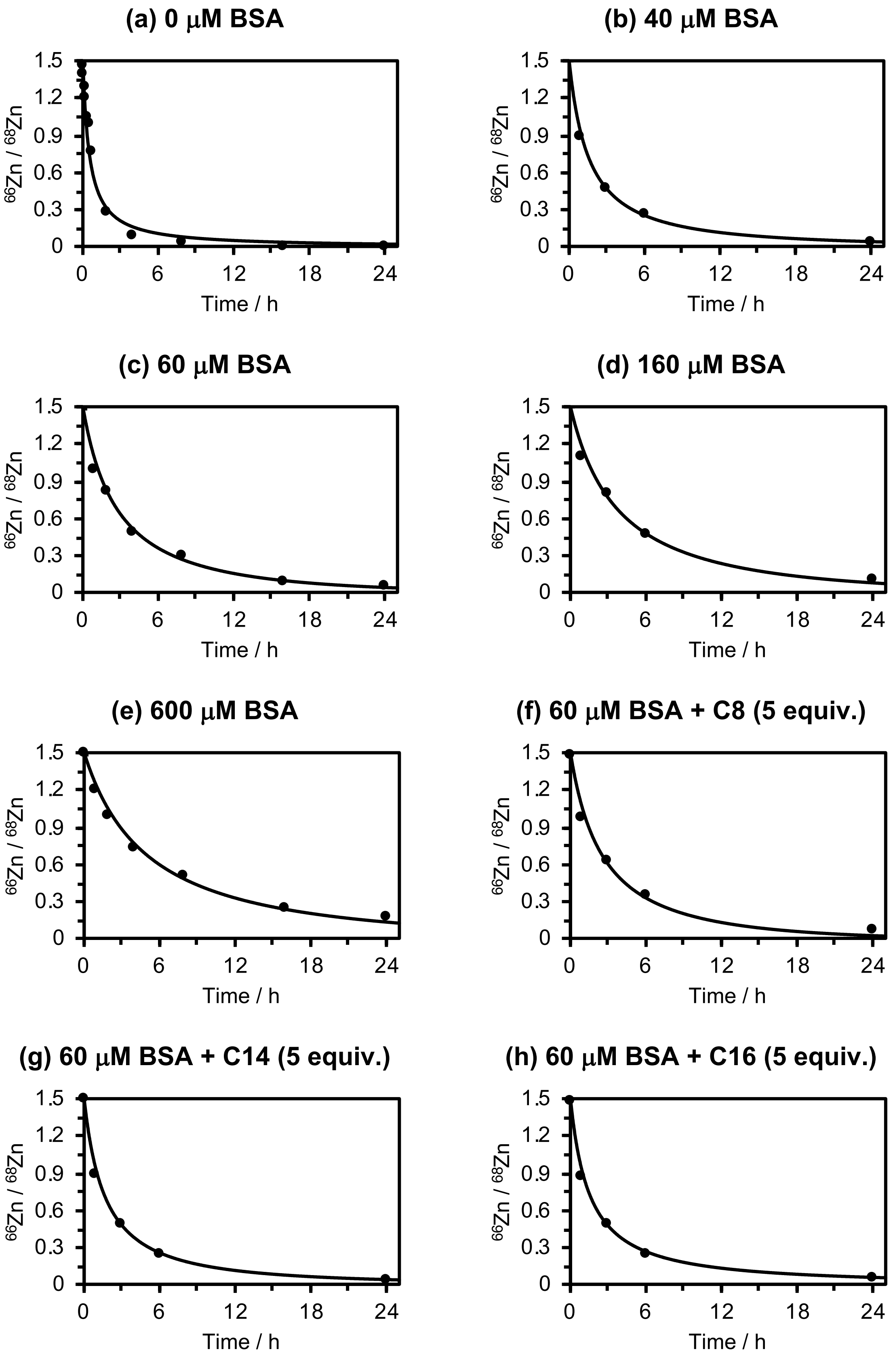

Figure S2. (a-h) Mathematical model fitting (curves) overlaid with experimental data (•) for the determined $^{66}Zn/^{68}Zn$ ratio over time in immortalised HUVECs. Fitting parameters and statistics: **Tables S8-S15**. Experimental data: **Tables S4** and **S6**.

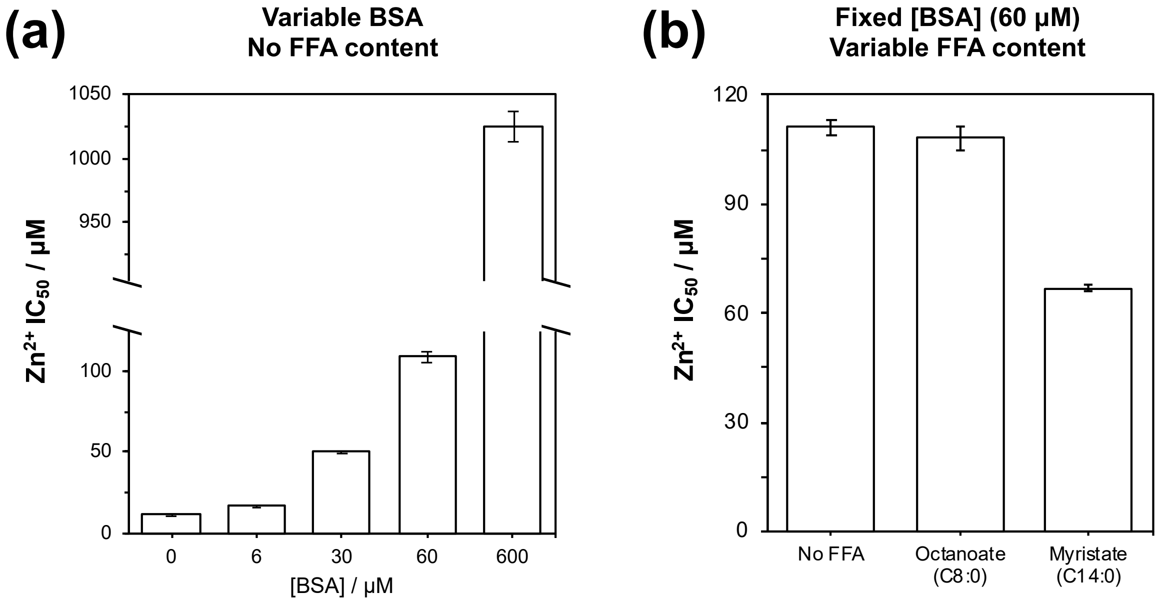

Figure S3. Zinc toxicity (IC_{50} / µM) determined for Zn^{2+} (as $ZnCl_2$) in immortalised HUVECs: (a) in the absence of FFAs with variable BSA concentration (0-0.6 mM); (b) with fixed extracellular BSA concentration (60 µM) in the presence or absence of fatty acids (FFAs): C8:0 octanoate, and C14:0 myristate. IC_{50} concentrations were determined in duplicate of triplicate experiments with cell viability determined using the Sulforhodamine B (SRB) assay.

Materials and Methods

Materials. Human umbilical vein endothelial cells (HUVECs) were immortalised using hTERT as described below and maintained in endothelial cell growth media (PromoCell GMBH, Germany). Earle's Balanced Salt Solution (EBSS; low $NaHCO_3$) was purchased from Lonza, UK. Cells were checked for mycoplasma-free status at 6-month intervals. Dulbecco's Modified Eagle Medium (DMEM), phosphate-buffered saline (PBS), Tris Base (pH 10, 1 mM) and Trypsin/EDTA solution (0.25%) were kindly prepared by technical services at the Department of Life Sciences (University of Warwick, UK). C-Chip Disposable hemocytometers were purchased from Labtech International. All other plastic products for cell culture were purchased from Greiner Bio-One (Gloucestershire, UK). Isotope-enriched ^{68}ZnO (>99%) was purchased from CK-Isotopes. Ultra-pure nitric acid (72% v/v) was freshly distilled before use. Milli-Q water (18.2 MΩ•cm at 298 K) was freshly purified by reverse osmosis before use. Acetonitrile (GC-MS grade), ammonium acetate (99.99% trace metals basis), ammonium hydroxide (28% NH_3; 99.99% trace metals basis), diisopropylethylamine, sulforhodamine B, zinc sulfate heptahydrate (99.995% trace metals basis), sodium octanoate, sodium myristate, sodium palmitate, bovine serum albumin (fatty acid-free) and all other reagents were purchased from Sigma Aldrich and used as received, unless indicated otherwise. Natural abundance Zn ICP-MS standard solution was purchased from Inorganic Ventures (CGZN1-1).

Immortalisation of primary human umbilical vein endothelial cells (HUVECs). Early passage primary HUVECs were obtained from TCS Cellworks (Buckinghamshire, UK) and grown in endothelial cell growth media containing supplement mix (complete media; PromoCell, Germany). HUVECs were grown to 70% confluency and transduced with supernatant from ψ-CRIPpBABEpurohTERT cells (as described in reference [1]) which were gifted by Prof. Andrew Riches (University of St Andrews). The ψ-CRIPpBABEpurohTERT cells were grown in DMEM supplemented with 2 mM glutamine and 10% foetal bovine serum (FBS) and reached confluency. The supernatant was collected, filtered (0.45 µm) and 10 µg mL^{-1} polybrene was added to enhance the binding of viral particles to the HUVEC membrane. Only a single transduction was performed. After 24 h, the supernatant was removed and HUVECs were given fresh complete medium. HUVECs were passaged and 0.2 µg mL^{-1} of puromycin was added to the medium for selection of cells.

Reverse transcription-polymerase chain reaction (RT-PCR). To establish the expression of endothelial markers (genes encoding CD32, von Willebrand factor and VE-cadherin) in the immortalised HUVECs, RNA was extracted using TRIzol reagent (Invitrogen, UK) according to the manufacturer's instructions. RNA was precipitated using isopropanol and quantified using by NanoVue (GE Healthcare, UK). A 2 µg aliquot of total RNA was reverse transcribed into cDNA using Oligo(dT)-15 primer (Promega, UK). One µL of the resultant cDNA sample was subjected to PCR analysis using Bio-X-ACT short mix (Bioline, UK) and 250 nM of each primer. The cycling profile was: 95°C for 30 s, 58°C for 30 s and 68°C for 30 s for 35 cycles, preceded

by a 2-minute enzyme activation at 95°C. Gene-specific primers for genes encoding CD31, vWF and VE-cadherin were designed and obtained from Eurofins Genomics (Ebersberg, Germany). PCR products were analysed on agarose gels composed of 1% (w/v) agarose, melted in Tris-acetate/EDTA-buffer (TAE-buffer). SyberSafe dye was added to the melted agarose to enable the detection of DNA in the gel. The gel was left to set and then was transferred into an electrophoresis tank containing TAE-buffer. Samples were prepared with a gel loading dye and Hyperladder I DNA-ladder was loaded (Bioline). Gels were run at 150 V for as long as required for the separation of bands. DNA was visualised in a Gel Doc XR+ Imager and analysed using Image Lab 2.0 software (Bio-Rad Laboratories, UK).

Preparation of isotope-enriched $^{68}Zn^{2+}$ stock solution. Isotope-enriched ^{68}ZnO was reacted with 2 molar equivalents of hydrochloric acid to obtain a solution of $^{68}ZnCl_2$. The pH of the stock solution was adjusted with HEPES buffer to achieve a final working pH = 7 and [Zn^{2+}] = 0.5 mM in 1× HEPES-buffered EBSS. The isotopic abundance of the stock solution was measured by ICP-MS (Agilent 7900 series ICP-MS in helium collision gas mode) and abundances are reported in **Table S1**.

Preparation of sterile isotopic-enriched $^{68}Zn^{2+}$ solution for cell culture. The previously described preparation of 0.5 mM $^{68}Zn^{2+}$ stock solution in buffered EBSS was diluted with Milli-Q doubly deionized water to achieve a final working concentration of 40 µM. This solution was combined in equal volumes with a solution prepared in HEPES-buffered EBSS containing BSA (in each case at 2× final working concentration). The resultant solution was filtered using a 0.2 µm sterile filter and incubated at 310 K for 24 h. Final working concentrations: 20 µM $^{68}Zn^{2+}$, 1× BSA (as required; see **Table 1** and **ESI Tables S2-S15**).

Zinc uptake experiments. Briefly, 5 × 10^6 HUVECs were seeded in P145 dishes using Endothelial cell growth medium (PromoCell, Germany) and incubated until achieving >90% confluence. (310 K, 5% CO_2 humidified atmosphere). The supernatant medium was then removed by aspiration, and cells were treated with physiological concentrations of BSA (600 µM) and natural abundance Zn^{2+} (20 µM) prepared in HEPES-buffered EBSS for 24 hours (NB the growth of human umbilical vein endothelial cells is contact-inhibited). Cells were then washed thoroughly (3×) with PBS, and then treated with test solutions: isotope-enriched $^{68}Zn^{2+}$ (20 µM) with defined bovine serum albumin supplementation (0-600 µM). Cell pellets were collected in a time-dependent manner using 1 mL Trypsin/EDTA (0.25%) and were re-suspended in PBS to obtain a single cell solution, from which a cell count was obtained using a hemocytometer. Cell pellets were obtained by centrifugation and repeated washing with PBS (3 × 1 mL). Cells were then re-suspended in 200 µL ultra-pure 72% nitric acid and digested at 351 K. After 24 h, solutions were diluted with milli-Q water to achieve a final working nitric acid concentration of 3.6% v/v (4 mL). Zinc content (^{64}Zn, ^{66}Zn, ^{67}Zn, ^{68}Zn, ^{70}Zn) was determined using an Agilent 7900 series ICP-MS in He-gas mode. The instrument was calibrated using freshly prepared Zn standard solutions (0.1-1000 ppb) in 3.6% v/v nitric acid

with an internal standard of ^{166}Er (50 ppb). Data were processed using MassHunter (Agilent Technologies, Inc., UK). Each timepoint is the result of three biological repeats. For the experiments involving FFAs, isotope-enriched $^{68}Zn^{2+}$ (20 μM) media were prepared in the presence of 60 μM BSA with 5 mol. equiv. (300 μM) of FFAs, octanoate (C8:0), myristate (C14:0) or palmitate (C16:0), which were pre-incubated at 310 K for 24 h. Fatty acids were not co-administered in the pre-conditioning step of any experiment.

Determination of Zn^{2+} toxicity. Briefly, 5×10^4 HUVECs were seeded per well in a 96-well plate using 0.15 mL of endothelial cell growth medium per well and incubated for 48 h (310 K, 5% CO_2 atmosphere). After this time, the supernatant was removed and cells were treated with defined concentrations of Zn^{2+} (as $ZnCl_2$, typically 0.001-10 mM) in HEPES-buffered EBSS, in the absence of FFAs and in the presence of either 0, 6, 30, 60, or 600 μM BSA for 24 h. After this time, the supernatant was removed, and cells were washed with PBS (2 × 200 μL). Cells were then fixed by addition of 50% trichloroacetic acid (50 μL per well) and incubated at 277 K for 1 h. The supernatant was removed by sequential washing with water, and cells were stained using sulforhodamine B (0.4% dye content in 1% acetic acid solution) for 30 min. After this time, excess dye was removed by sequential washing with 1% acetic acid solution, and then dye was liberated by addition of Tris base (pH 10.5, 10 mM, 200 μL per well) for 1 h. Absorbance was measured using a Thermo Scientific SkanIt microplate reader fitted with a 492 nm filter. Absorbance measurements were normalized relative to untreated negative control wells to determine cell survival. IC_{50} value determinations were carried out as duplicate of triplicate experiments, and the IC_{50} values were calculated using Origin for Windows to fit sigmoidal curves to experimental data. Data are reported as the average of each duplicate experiment with associated standard deviation. The experiment was repeated with the following modification: Zn^{2+} toxicities were determined as described above using a fixed concentration of 60 μM bovine serum albumin (equivalent to 10% fetal calf serum routinely used in cell culture experiments) in the presence of FFA (0.3 mM, 5 mol. equiv., C8:0 octanoate or C14:0 myristate) for 24 h.

Calculation of free zinc concentrations. Free [Zn^{2+}] was estimated based on affinity data for the primary binding site, namely stoichiometric stability constants of log K = 7.0 and = 7.6.[2, 3] Using a published pK_a = 8.2,[2] these were converted to conditional stability constants valid at pH 7.4, giving log K' = 6.13 and 6.73. These were then used, together with total BSA and Zn^{2+} concentrations, to calculate free [Zn^{2+}], for both constants separately, to give an upper and lower limit. The data plotted in Figure 3(b) are the average of these two values. It may be noted that the resulting concentrations are likely an over-estimate, as they do not take binding to site B into account. We have refrained from attempting to include site B, as no stoichiometric or suitable conditional stability constant is available for this site.

Derivation of the Mathematical Model

Let $Q(t)$ denote the cellular zinc content (expressed in fg per cell). This zinc is present as various isotopes, denoted by a subscript $\star$, where $\star \in \{64,66,67,68,70\}$. These isotopes together constitute the total:

$$Q(t) = \sum_{\star \in \{64,66,67,68,70\}} Q_\star$$

The rate of change of $Q(t)$ is given by the difference between the influx ϕ_{in} and the efflux ϕ_{out}:

$$\frac{\mathrm{d}}{\mathrm{dt}} Q(t) = \phi_{\mathrm{in}} - \phi_{\mathrm{out}} \; .$$

We assume that fluxes are pro rata, that is $\phi_{\star,\mathrm{in}}(t) = \mathrm{r}_{\star,\mathrm{ex}} \phi_{\mathrm{in}}(t)$ where $\mathrm{r}_{\star,\mathrm{ex}} \in [0,1]$ is the relative abundance of isotope $\star$ in the extracellular medium, and similarly, $\phi_{\star,\mathrm{out}} = \mathrm{r}_{\star,\mathrm{cell}} \phi_{\mathrm{out}}$ where $\mathrm{r}_{\star,\mathrm{cell}} \in [0,1]$ is the relative abundance of isotope $\star$ inside the cells. We assume that $\mathrm{r}_{\star,\mathrm{ex}}$ remains constant for the duration of the experiment for all isotopes. This assumption is reasonable since the total extracellular quantity of zinc, circa 13 µg, exceeds the quantity contained in biomass by a factor of at least 30. By contrast, cellular abundances are changing over time as a result of influx (and efflux):

$$\mathrm{r}_{\star,\mathrm{cell}}(t) = \frac{Q_\star(t)}{Q(t)}$$

Furthermore, these changing cellular abundances are observed in the experiments which focus on the ratio $^{66}Zn/^{68}Zn$:

$$\frac{^{66}\mathrm{Zn}}{^{68}\mathrm{Zn}}(t) = \frac{\mathrm{r}_{66,\mathrm{cell}}}{\mathrm{r}_{68,\mathrm{cell}}}(t) = \frac{Q_{66}(t)/Q(t)}{Q_{68}(t)/Q(t)} = \frac{Q_{66}(t)}{Q_{68}(t)}$$

The pro rata assumption yields the kinetics for each isotope:

$$\frac{\mathrm{d}}{\mathrm{dt}} Q_\star(t) = \phi_{\star,\mathrm{in}}(t) - \phi_{\star,\mathrm{out}}(t) = \mathrm{r}_{\star,\mathrm{ex}} \phi_{\mathrm{in}}(t) - \mathrm{r}_{\star,\mathrm{cell}}(t) \phi_{\mathrm{out}}(t)$$

$$= \mathrm{r}_{\star,\mathrm{ex}} \phi_{\mathrm{in}}(t) - \frac{Q_\star(t)}{Q(t)} \phi_{\mathrm{out}}(t) \; .$$

Applying the quotient rule and substituting the above expressions for the rates of change of $Q(t)$ and $Q_\star(t)$, we find:

$$\frac{\mathrm{d}}{\mathrm{dt}} \frac{Q_\star(t)}{Q(t)} = \frac{\phi_{\mathrm{in}}(t)}{Q(t)} \left(\mathrm{r}_{\star,\mathrm{ex}} - \frac{Q_\star(t)}{Q(t)} \right) .$$

It is useful to switch to the auxiliary variable

$$W_\star(t) = r_{\star,ex} - Q_\star(t)/Q(t)$$

as this variable satisfies a particularly simple differential equation:

$$\frac{d}{dt}W_\star(t) = -\frac{\phi_{in}(t)}{Q(t)}W_\star(t)$$

the solution of which is a standard formula:

$$W_\star(t) = W_\star(t_0)\exp\left\{-\int_{t_0}^{t}\frac{\phi_{in}(\tau)}{Q(\tau)}d\tau\right\}.$$

This yields the following general solution for the observed isotope ratio:

$$\frac{^{66}Zn}{^{68}Zn}(t) = \frac{r_{66,ex} - W_{66}(t_0)G(t)}{r_{68,ex} - W_{68}(t_0)G(t)}$$

with

$$G(t) = \exp\left\{-\int_{t_0}^{t}\frac{\phi_{in}(\tau)}{Q(\tau)}d\tau\right\}.$$

The discussion to this point is valid even when the fluxes vary with time, but we now assume that they are constant over the duration of the experiment (*vide infra* for a justification of this assumption). We then have:

$$Q(t) = Q(t_0) + (\phi_{in} - \phi_{out})(t - t_0),$$

and with this, the integral in $G(t)$ can be evaluated to give:

$$G(t) = \left(1 + \frac{(\phi_{in} - \phi_{out})}{Q(t_0)}(t - t_0)\right)^{-\phi_{in}/(\phi_{in} - \phi_{out})}.$$

In the special case where $\phi_{in} = \phi_{out} \equiv \phi$ we have $Q(t) \equiv Q_0$, a condition we refer to as flux-balanced; in this case, $G(t)$ reduces to

$$G(t) = \exp\{-(\phi/Q_0)(t - t_0)\}.$$

We now identify time $t = t_0$ as the start of the incubation experiment, and we assume that at this point in time, the cellular isotope abundances are equal to the natural ones, which we denote with a tilde. Recalling the definition of $W_\star$, we have:

$$\frac{^{66}Zn}{^{68}Zn}(t) = \frac{r_{66,ex} - (r_{66,ex} - \tilde{r}_{66})G(t)}{r_{68,ex} - (r_{68,ex} - \tilde{r}_{68})G(t)}.$$

The observed ratio thus shifts from $\tilde{r}_{66}/\tilde{r}_{68}$ at time t_0 to $r_{66,ex}/r_{68,ex}$ as $t\to\infty$.

Model selection and non-linear least-squares fitting. Prior to modelling the $^{66}Zn/^{68}Zn$ ratio data, linear regression was used to assess whether the cells accumulated a statistically

significant amount of zinc during the experiment. If the null hypothesis of a slope equal to zero could not be rejected, the cells did not change their total content, meaning that flux-balance was given, and the equations valid for $\phi_{\mathrm{in}} = \phi_{\mathrm{out}} \equiv \phi$ were used. The influx was estimated by non-linear least-squares fitting of the data to the model given above; for the flux-balanced case, Q_0 was set equal to 52 fg·cell^{-1}. If the null hypothesis was rejected, the parameters ϕ_{in} and ϕ_{out} were estimated by non-linear least-squares fitting of the $^{66}Zn/^{68}Zn$ data to the general model given above.

Non-linear least-squares curve fitting was carried out using `NonlinearModelFit` in Mathematica. A minimum was located, except in the [BSA] = 0 µM experiment, where the estimates for ϕ_{in} and ϕ_{out} converged to a common value in spite of clear evidence that $\phi_{\mathrm{in}} > \phi_{\mathrm{out}}$. To obtain a parameter estimate in this case, the quantity $(\phi_{\mathrm{in}} - \phi_{\mathrm{out}})$ was set equal to the estimated slope of the linear regression to the accumulation data, and only ϕ_{in} was subsequently estimated via the normal procedure.

Justification for assuming constant fluxes. To validate the assumption that fluxes were constant over time, an empirical reconstruction approach was used. Briefly, we have the following explicit expressions for the fluxes as a function of time:

$$\phi_{\mathrm{in}}(t) = -Q(t)\frac{G'(t)}{G(t)}$$

and

$$\phi_{\mathrm{out}}(t) = -Q(t)\frac{G'(t)}{G(t)} - Q'(t)$$

with

$$G(t) = \frac{\mathrm{r}_{66,\mathrm{ex}} - \mathrm{r}_{68,\mathrm{ex}}R(t)}{\mathrm{r}_{66,\mathrm{ex}} - {}_{66,\mathrm{ex}} - \left(\mathrm{r}_{68,\mathrm{ex}} - \tilde{\mathrm{r}}_{68,\mathrm{ex}}\right)R(t)}$$

where $R \equiv {}^{66}Zn/^{68}Zn$. This means that the fluxes can in fact be reconstructed as a function of time if R and Q have been sampled sufficiently frequently in time, with sufficient accuracy, to warrant the fitting of a suitable smooth curve. A drawback is that the expressions involve differentiation, which tends to exacerbate the effects of any inaccuracies. However, this approach was merely taken to test whether the assumption of time-constant influx and efflux over the course of the experiment was warranted. By way of representative example with relatively high influx and non-constant $Q(t)$, let us consider the accumulation and isotope ratio data for the [BSA] = 40 µM experiment. The figure below shows smooth curve fits to the data, giving $R(t)$ and $Q(t)$ empirically, as well as the resulting reconstructed fluxes. The latter are broadly consistent with the assumption of time-constant fluxes, although there is evidence that the efflux experiences a transient at the beginning of the experiment.

The assumption of constant fluxes is an idealisation that results in excellent goodness of fit, whereas the empirical-reconstructive approach suggests that the cells go through an initial period of acclimatisation. Inasmuch as the data do not clearly favour one or the other

hypothesis, we have preferred to adhere to the simplicity of the mechanistic analysis presented in the main text.

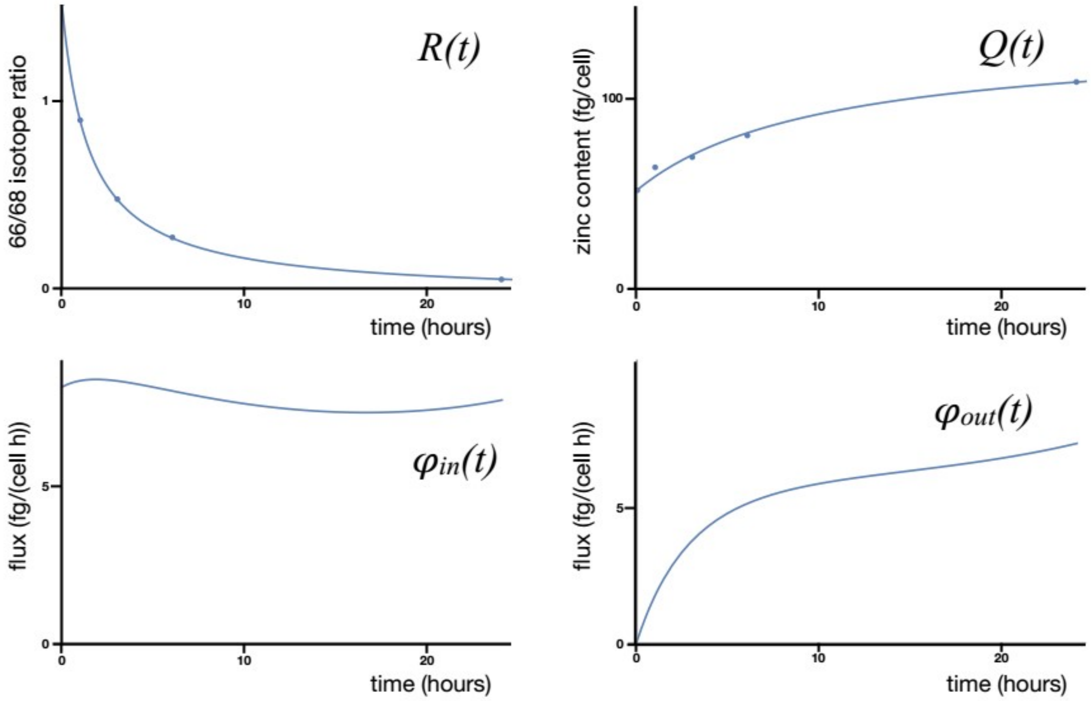

References

1. R. D. Ramirez, S. Sheridan, L. Girard, M. Sato, Y. Kim, J. Pollack, M. Peyton, Y. Zou, J. M. Kurie, J. M. Dimaio, S. Milchgrub, A. L. Smith, R. F. Souza, L. Gilbey, X. Zhang, K. Gandia, M. B. Vaughan, W. E. Wright, A. F. Gazdar, J. W. Shay and J. D. Minna, *Cancer Res.*, 2004, **64**, 9027-9034.
2. E. Ohyoshi, Y. Hamada, K. Nakata and S. Kohata, *J. Inorg. Biochem.*, 1999, **75**, 213-218.
3. J. Lu, A. J. Stewart, D. Sleep, P. J. Sadler, T. J. T. Pinheiro and C. A. Blindauer, *J. Am. Chem. Soc.*, 2012, **134**, 1454-1457.
